# Cerebellar correlates of social dysfunction among individuals at clinical high risk for psychosis

**DOI:** 10.3389/fpsyt.2022.1027470

**Published:** 2022-12-01

**Authors:** Isabelle R. Frosch, Katherine S. F. Damme, Jessica A. Bernard, Vijay A. Mittal

**Affiliations:** ^1^Department of Psychology, Northwestern University, Evanston, IL, United States; ^2^Institute for Innovations in Developmental Sciences, Northwestern University, Evanston, IL, United States; ^3^Department of Psychological and Brain Sciences, Texas A&M University, College Station, TX, United States; ^4^Texas A&M Institute for Neuroscience, Texas A&M University, College Station, TX, United States; ^5^Department of Psychiatry, Northwestern University, Chicago, IL, United States; ^6^Department of Medical Social Sciences, Northwestern University, Chicago, IL, United States; ^7^Institute for Policy Research, Northwestern University, Chicago, IL, United States

**Keywords:** cerebellum, social functioning, clinical high risk (CHR) for psychosis, prodrome, resting state

## Abstract

**Introduction:**

Social deficits are a significant feature among both individuals with psychosis and those at clinical high-risk (CHR) for developing psychosis. Critically, the psychosis risk syndrome emerges in adolescence and young adulthood, when social skill development is being fine-tuned. Yet, the underlying pathophysiology of social deficits in individuals at CHR for psychosis remains unclear. Literature suggests the cerebellum plays a critical role in social functioning. Cerebellar dysfunction in psychosis and CHR individuals is well-established, yet limited research has examined links between the cerebellum and social functioning deficits in this critical population.

**Method:**

In the current study, 68 individuals at CHR for developing psychosis and 66 healthy controls (HCs) completed social processing measures (examining social interaction, social cognition, and global social functioning) and resting-state MRI scans. Seed-to-voxel resting-state connectivity analyses were employed to examine the relationship between social deficits and lobular cerebellar network connectivity.

**Results:**

Analyses indicated that within the CHR group, each social domain variable was linked to reduced connectivity between social cerebellar subregions (e.g., Crus II, lobules VIIIa and VIIIb) and cortical regions (e.g., frontal pole and frontal gyrus), but a control cerebellar subregion (e.g., lobule X) and was unrelated to these social variables.

**Discussion:**

These results indicate an association between several cerebellar lobules and specific deficits in social processing. The cerebellum, therefore, may be particularly salient to the social domain and future research is need to examine the role of the cerebellum in psychosis.

## Introduction

Social deficits appear early during the clinical high-risk period (CHR) for developing psychosis and persist throughout the clinical course (1, 2). Poor social abilities predict poor clinical and functional outcomes for both individuals at CHR for psychosis and those with a psychosis diagnosis (1, 3–7). Therefore, the social domain may be a powerful treatment target that could impact disease course and functional outcomes. Treatments targeting social deficits are limited as the neural mechanisms underlying them are poorly understood in this population. Parallel work in other populations [i.e., autism spectrum disorder (ASD)] and in healthy social processing suggests that the cerebellum may play a critical role in social ability (8). Yet, this possibility has not been explored in individuals at CHR for psychosis.

Three crucial components of social ability particularly relevant to the psychosis spectrum are: social cognition, social interaction, and social functioning. Social skills represent the dynamic and synchronous combination of complex behaviors. Competency in one social skill can give rise to another. For example, the ability to engage in a social interaction successfully relies on the ability to accurately appraise and respond to social situations. Together, the ability to conduct successful social interactions and apply appropriate social cognition, is paramount to building and maintaining a strong and extensive social network. It is important to consider broad and specific social skills given their complexity and interrelated nature. Here, the three primary social abilities of are imagined as interrelated layers, where social cognition is a core tenet of successful interactions, which then enables and reinforces successful social functioning. An extensive literature has already identified numerous facets of social processing impairment across the psychosis spectrum, yet their underlying biological mechanisms remain unclear (9–17). Understanding these mechanisms is fundamental for isolating specific treatment targets.

Biological mechanisms of social impairments in the CHR period for psychosis can provide critical information about the nature of social deficits. The neurobiology of social cognition among individuals at CHR for psychosis implicates cortical regions including anterior cingulate cortex (ACC), superior temporal gyrus (STG), medial prefrontal cortex (MPFC), orbitofrontal cortex (OFC), postcentral gyrus, supramarginal gyrus (SMG), insula, and temporoparietal junction (TPJ) (18–21). Extant work primarily focuses on cortical regions indicated in emotional processing and has not considered the contributions of subcortical regions associated with similar social deficits in other clinical populations.

Social difficulties experienced by autistic individuals^1^ and those with psychotic disorders are markedly similar (23–26). Despite the clinical similarity, neuroimaging work in these respective disorders considers separate neural correlates to social challenges. The cerebellum is the primary and most consistent site of ASD-related symptoms, particularly social behaviors (27–29). Although the psychosis field has predominantly focused on cortical and limbic regions implicated in social deficits (20, 30, 31). Cognitive and sensorimotor research indicates a central role of the cerebellum in psychosis and psychosis risk, its contribution to social deficits remains largely unexplored. To date, most studies of psychosis and individuals at CHR for psychosis are focused on cerebellar contributions to motor abnormalities, timing abnormalities, predictive learning, and symptom severity within the psychosis domain (32–36).

The current study investigated cerebellar resting-state functional connectivity within the context of social deficits (social interaction, cognition, and function) in those at CHR for psychosis syndrome. A multidimensional approach was applied to capture the richness of social processing by including retrospective parental observation, clinical assessment ratings, and a test of social cognitive processing. We predict aberrant connectivity is linked to social functioning deficits, and test this by examining group differences in the interaction between connectivity in social cerebellar regions and social function metrics (37–41). Furthermore, based on literature in the general population (42), as well as clinical populations [e.g., autism, schizophrenia (43, 44)], we predict that when compared to healthy controls, CHR individuals will have aberrant connectivity stemming from socially-mediated areas of the cerebellum (i.e., posterior lobules). We hypothesize that these predicted functional neural deficits in the CHR group will be associated with social deficits in domains of social interaction, cognition, and overall functioning. To assess whether impairments in social domains were tied to specific cerebellar social lobule abnormalities, we examined links with social processes and a control region (lobule X, which is implicated in vestibular control). We would not expect this region to be associated with social abilities. Given the breadth of social deficits found among individuals at CHR for psychosis, we expect that each social domain will be linked with cerebellar abnormalities. By exploring distinct relationships between these social domains and the cerebellum, we aim to shed light on what is and is not contributing to these social deficits, which might help to guide future research and intervention.

## Methods

### Participants

A total of 134 adolescents and young adults 68 CHR, 66 HC were enrolled in the Adolescent Development and Preventive Treatment (ADAPT) Program. CHR status was determined by the presence of attenuated psychosis symptoms, or the presence of schizotypal personality disorder accompanied by a global functioning decline at or before the age of 19, or a family history of psychosis with global functioning decline. Participants were excluded if they met any of the following: younger than 14 or older than 24, diagnosed with a psychotic disorder, diagnosed with ASD, history of traumatic head injuries or neurological disorders, a lifetime history of substance abuse disorder, contraindications for MRI. All participants provided written informed consent/assent (in the case of minors, guardians provided informed consent) and were compensated for their time. All procedures were approved by the University Institutional Review Board.

Subsamples of these participants have been evaluated with respect to non-motor learning rules (45), cerebellar contributions to symptom severity (46), abnormal hippocampal shape and symptom progression (47), postural sway abnormalities related to cerebellum dysfunction (32), sleep dysfunction (48), and emotion recognition (49). The current study is the first analysis of cerebellar subregions implicated in social cognition that has been conducted or any of the three primary social outcome variables have been analyzed in this sample.

#### Clinical characterization

Psychodiagnostic interviews were administered by trained assessors and included the Structured Interview for Prodromal Syndromes (SIPS) (50) to determine the presence of attenuated psychosis symptoms and the Structured Clinical Interview for DSM-IV (SCID) (51) to rule out psychosis, substance abuse, and diagnose other psychiatric disorders.

### Measures of social abilities

#### Social interaction

The Autism-Tics, ADHD and other Comorbidities inventory (A-TAC) (52) was used to assess retrospective social interaction quality. The A-TAC is a parent-informed questionnaire intended to identify broad phenotypic indicators of neurodevelopmental and psychiatric diagnoses across a child's lifetime. There are five items in the social interaction subscale rated as follows, “No” scored as 0, “Yes, to some extent” scored as 0.5, and “Yes” scored as 1. Total subscale scores are calculated by summing each item such that a maximum score of 5 indicates deficient reciprocal social behaviors and 0 indicates no social interaction issues.

#### Social cognition

Social cognition was assessed using the Managing Emotions subtest of the Mayer-Salovey-Caruso Emotional Intelligence Test (MSCEIT-ME) (53). In this neuropsychological assessment, participants are presented with 8 brief vignettes of difficult social situations and then four possible reactions, each of which varies in appropriate levels of emotional reactivity. Participants are instructed to rate the four reactions based on social effectiveness using a 5-point scale from 5, which would be very ineffective to 1, very effective. This scale was developed for populations with schizophrenia or other severe mental illness and has been successfully implemented in early high-risk populations (11).

#### Social functioning

Clinical impressions of current social functioning were made throughout the clinical interviews and assessed using the Global Functioning Scale—Social; GFS-S (54). The GFS-S evaluates the quality and quantity of peer relationships, peer conflict, age-appropriate romantic relationships, and relationships with family members were evaluated. Assessors provide an overall score ranging from 1 to 10, with 1 indicating severe social impairment and 10 indicating superior social functioning. The GFS-S has been widely used in clinical samples including participants at CHR for developing psychosis and has been shown to have strong internal consistency (55).

### Image acquisition

Every participant completed a structural and resting-state functional scan acquired on a 3-Tesla Siemens Tim Trio MRI scanner (Siemens, AG, Munich, Germany), using a standard 12-channel head coil. First, structural images were collected with a T1-weighted 3D magnetization prepared rapid gradient multi-echo sequence [MPRAGE; sagittal plane; repetition time (TR) = 2,530 ms; echo times (TE) = 1.64, 3.5, 5.36, 7.22, and 9.08 ms; GRAPPA parallel imaging factor of 2; 1 mm 3 isomorphic voxels, 192 interleaved slices; FOV = 256 mm; flip angle = 7°; time = 6:03 min]. This was followed by a resting-state blood-oxygen-level-dependent (BOLD) scan during which participants were asked to close their eyes and relax. The scan was collected with a T2-weighted echo-planar functional protocol (number of volumes = 165; TR = 2,000 ms; TE = 29 ms; matrix size = 64 × 64 × 33; FA = 75°; 3.8 × 3.8 × 3.5 mm 3 voxels; 33 slices; FOV = 240 mm; time = 5:34 min).

### MRI scanning procedure

A turbo spin echo proton density (PD)/T2-weighted acquisition (TSE; axial oblique aligned with anterior commissure-posterior commissure line; TR = 3,720 ms; TE = 89 ms; GRAPPA parallel imaging factor of 2; FOV = 240 mm; flip angle: 120°; .9x.9 mm voxels; 77 interleaved 1.5 mm slices) was acquired to check for incidental pathology.

### Resting state functional magnetic resonance imaging preprocessing

Data were preprocessed in FSL (v.5) (56–58), which involved motion correction, brain extraction, high-pass filtering (100 s), and spatial smoothing (6 mm FWHM). Next, functional images were aligned to the MNI 2-mm brain template with a two-step procedure. In the first step, the resting-state scan was aligned to the high-resolution MPRAGE using a linear boundary-based registration method, which relies on white matter boundaries (59). For the second step, the MPRAGE was non-linearly aligned to the template and the two registrations were then combined to align the fcMRI scan to the template. To account for motion-related artifacts, temporal and motion derivative regressors were calculated with the Artifact Rejection Toolbox (ART; http://www.nitrc.org/projects/artifact_detect/) for both outliers based on mean signal (>3 SD) and motion (>1 mm total). The resultant motion regressors were entered into the model as a temporal derivative nuisance covariate at the subject level.

### Motion-related artifact control details

To account for motion-related artifacts, temporal derivative regressors were calculated with the Artifact Rejection Toolbox (ART; http://www.nitrc.org/projects/artifact_detect/). This resulted in three translation and three rotation parameters with additional image specific confound regressors based on brain activation and framewise movement. Brain activation outliers were calculated using both the mean global brain activity and z-normalized mean signal across all voxels as a function of time. Outliers were defined as any frames where the global mean signal exceeded 3 standard deviations. Framewise measures of motion (composite measure of total motion, or maximum voxel displacement, across translation and rotation) were used to identify any motion outliers. Motion outliers were defined as frames where the absolute value of motion exceeded 1 mm. The resultant motion regressors were entered into the model as a temporal derivative nuisance covariate at the subject level. Independent *t*-tests were used to examine group differences in total mean signal and motion outliers. Results indicated there were no significant group differences in the number of signal outliers *t*_(−0.625)_ = 132, *p* = 0.533. There was a trending difference in motion outliers where individuals at CHR for psychosis had fewer compared to their HC peers *t*_(−1.95)_ = 132, *p* = 0.054.

### Functional connectivity: Statistical analyses

Functional connectivity analyses were performed in the CONN toolbox v20.b (60) and SPM12. The data were band-pass filtered from 0.008 to 0.09 Hz. Anatomical images were segmented into gray matter, white matter, and CSF with SPM12 in order to create masks for signal extraction. Five temporal components from segmented CSF and white matter were extracted using a principal components analysis within the CONN toolbox. These were used to correct for motion and physiological noise without regressing out global signal, thus allowing for equivalent global signal.

Regions of interest (ROIs), including the bilateral posterior cerebellum (lobules VIIa, VIIb, VIIIa, and VIIIb), bilateral Crus II and bilateral Lobule X, were defined based on the SUIT atlas (61, 62). Posterior cerebellum and Crus II have been shown to contribute to higher-order cognition in the cerebellum in social abilities (42, 63–65). To assess specificity across the cerebellar lobule ROIs, lobule X, which is primarily involved in vestibular functions, was used as a control region. The mean time-series, averaged across all voxels within each lobular ROI, was used as regression coefficient. It was then correlated with all other voxels in the brain in separate seed-to-voxel connectivity analyses for each ROI. We completed a model for each ROI to investigate relationships between connectivity and the scores on the three measures of social function. All analyses were conducted as interactions such that we investigated areas where the associations between seed-to-voxel connectivity and scores on the measure of interest were different between the CHR and control groups. Therefore, analyses yielded only the regions identified in the results and Supplementary Table 1. Results were thresholded at *p* < 0.001 at the voxel-level, with a false discovery rate (FDR) cluster-level correction of *p* < 0.05 (66). To control for the number of social measurements, a Bonferroni adjusted alpha level of 0.017 (0.5/3) was applied to each ROI analysis. To control for outliers further, we applied a robust linear regression to these data using the MASS R statistical package (67).

### Demographic analyses

Demographic data and behavioral differences were assessed using independent samples *T*-tests and Chi-squared tests using SPSS, v27. In three separate models, social interaction, social cognition, and social functioning were compared across diagnostic groups (CHR and HC) using *t*-tests. All analyses (imaging and group comparisons on social measures) were run with and without participants using antipsychotics and given that there was no difference in findings when omitting these participants (*n* = 8), we included them in subsequent analyses. *Post-hoc* analyses also controlled for sex and there was no significant group by sex interaction in any of the connectivity analyses.

## Results

### Demographic characteristics

Participant demographic and clinical characteristics are summarized in Table 1. There was no difference in age, or parental education, however, there was a difference between groups in sex, whereby the CHR group had significantly more males than females and the HC group had more females than males, $X_{\left(1\right)}^{2}$ = 5.02, *p* = 0.03. There were no group differences by sex in any of the connectivity analyses.

**Table 1:** Demographic and social functioning characteristics.

	**CHR (*n* = 68)**	**HC (*n* = 66)**	**Total sample (*n* = 134)**	**Statistic**	***P***
Age mean (SD)	18.78 (1.52)	18.79 (1.90)	18.78 (1.71)	*t*_(132)_ = −0.03	0.977
**Biological sex**
Female	38.2%	57.6%	46.8%	$X_{\left(1\right)}^{2}$ = 5.02	0.03
Male	61.8%	42.4%	52.2%		
Caregiver education (years) mean (SD)	15.41 (3.02)	15.64 (2.89)	15.52 (2.95)	*t*_(127)_ = −0.46	0.655
Current antipsychotic use (%)	11.8%	*na*			
Social interaction	*N* = 46	*N* = 28	*N* = 74		
	1.17 (1.24)	0.36 (0.55)	0.87 (1.10)	*t*_(72)_ = 3.21	<0.001
Social cognition	*N* =65	*N* = 63	*N* = 128		
	44.92 (9.7)	48.90 (8.83)	46.88 (9.50)	*t*_(126)_ = −2.42	0.17
Social functioning	*N* = 68	*N* = 65	*N* = 133		
	6.54 (1.77)	8.66 (0.69)	7.58 (1.72)	*t*_(133)_ = −8.99	<0.001

Parental education is the average years of education across both parents. Social interaction refers to social interaction difficulties measured by the Autism-Tics, ADHD and other Comorbidities inventory (A-TAC) (52) in which higher scores refer to increased social interaction impairment. Social cognition is quantified by the Managing Emotions subtest of the Mayer-Salovey-Caruso Emotional Intelligence Test [MSCEIT-ME; (53)] higher scores reflect efficient social cognition. Social functioning scores were tabulated using the Global Functioning Scale – Social (GFS-S) (54), where higher scores indicate successful maintenance, quality, and quantity of social relationships. - refers to negative t-value.

### Group differences in social deficits

**Figure 1:**
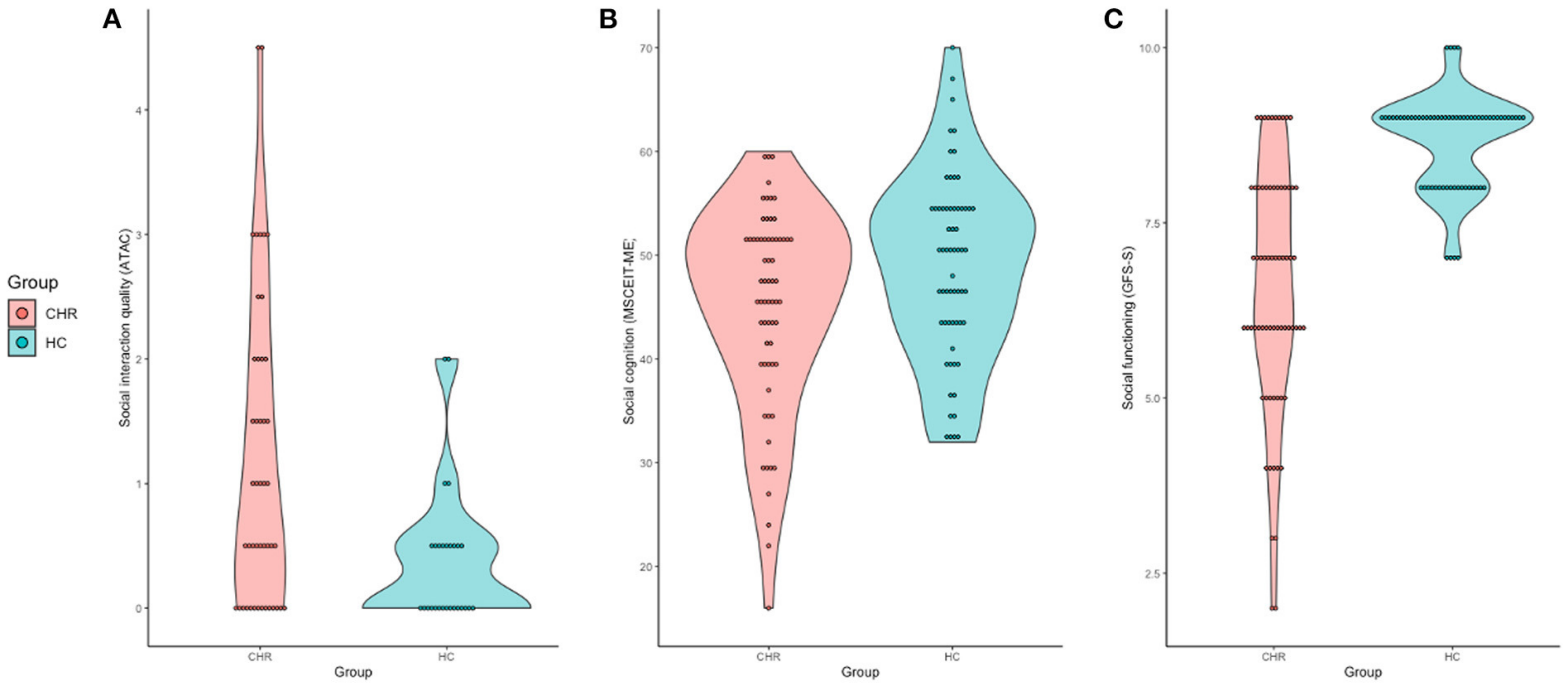
Violin plots of group differences between behavioral social ability measures. **(A)** Social interaction quality, measured by the ATAC plotted by group. **(B)** Social cognition scores as measured by the MSCEIT-ME plotted by group. **(C)** Social functioning ability, measured by the GFS-S plotted by group.

As expected, the CHR group demonstrated impaired social function compared to controls; ranging from retrospective accounts of social interaction quality [*t*_(74)_ = 3.42, *p* = 0.001], clinical impression of social functioning [*t*_(140)_ = −9.19, *p* < 0.001], and social cognition [*t*_(132)_ = −2.40, *p* = 0.018] (Figure 1).

### Connectivity patterns in social processing by group

Connectivity analyses were conducted to examine the role of posterior cerebellar lobules in social processing and identify potential mechanistic differences between the two groups related to social deficits. To assess the relationship between social interaction, social cognition, social functioning on cerebellar connectivity across CHR and HC groups, a mean-centered social covariate was compared across groups to predict any connectivity effect of social cerebellar regions (lobules VIIa, VIIb, VIIIa, and VIIIb), bilateral Crus II) in separate models. To control for the three social regions, a Bonferroni correction was applied (only FDR-corrected values of less or equal to 0.017 (0.05/3) were considered significant. Analyses were run for each of the three social measurements. Below includes the significant result from these analyses, see Supplementary material for details about trending results related to social interaction quality and social cognition. Given the demographic sex differences between groups, sex was added as a covariate across all connectivity analyses and did not change the magnitude or direction of findings.

#### Social interaction

To assess the relationship between social interaction quality on cerebellar connectivity across CHR and HC groups, a mean-centered social interaction covariate was compared across groups to predict any connectivity effect of lobule VIIIa. No group by social interaction associated with connectivity survived correction for multiple comparisons (Supplementary Table 2).

#### Social cognition

To assess the relationship between social cognition on cerebellar connectivity across CHR and HC groups, a mean-centered social covariate was compared across groups to predict any connectivity effect on social cerebellum regions (lobules VIIa, VIIb, VIIIa, and VIIIb), bilateral Crus II) in separate models. Similarly, after the Bonferroni correction for multiple comparisons, there was not a significant interaction between group by social cognition by connectivity (Supplementary Table 2).

#### Social functioning

**Figure 2:**
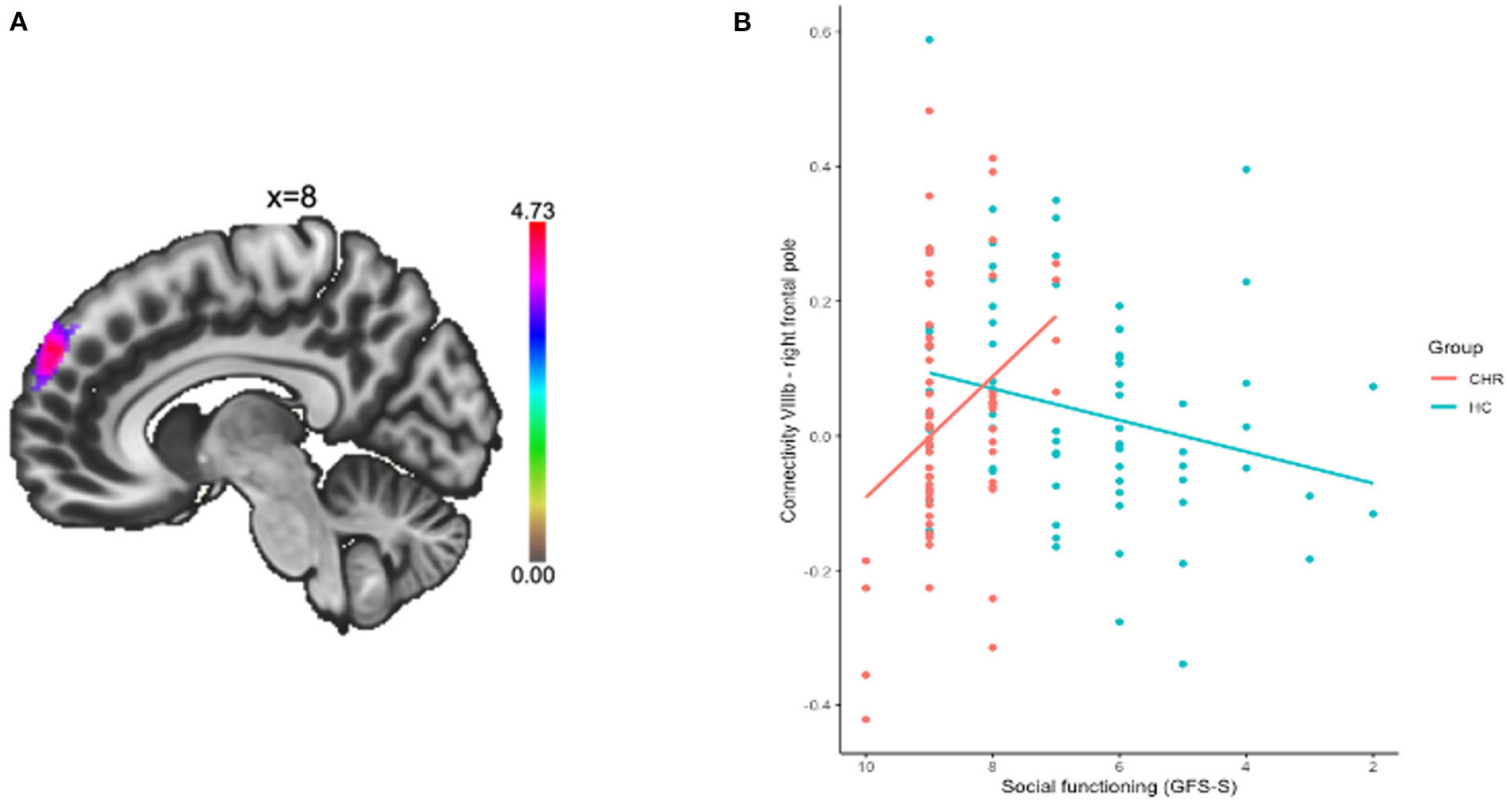
Connectivity between lobule VIIIb and right frontal pole was related to social functioning. **(A)** Cluster in the right frontal pole is depicted. **(B)** Connectivity between the regions was extracted and plotted by group and social functioning quality.

A mean-centered social functioning covariate was used to compare patterns of cerebellar connectivity and social functioning quality between groups. There was a significant group by social functioning interaction associated with connectivity between VIIIb and right frontal pole (*p*_*FDR*_ = 0.002). When this model was fit *via* a robust regression, which is less sensitive to outliers than ordinary least squares, we find a two-tailed *p*-value of 0.019 for social functioning (see Table 2; Figure 2). Lower connectivity between lobule VIIIb and right frontal pole related to poor social functioning in the CHR group (*r* = 0.30, *p* = 0.015). The opposite pattern was shown in the HC group wherein higher connectivity between lobule VIIIb and right frontal pole related to higher social functioning scores (*r* = −0.29, *p* = 0.024).

**Table 2:** Cerebellar seed to voxel connectivity analysis.

	**Coordinates**			
	**x**	**y**	**Z**	**cluster size**	***p_*FDR*−*corrected*_***	***p_*uncorrected*_***
**Social functioning**
VIIIb – right frontal pole	+10	+54	+32	268	0.002	0.0001

A Bonferroni correction was applied to control for the three social regions. Only FDR-corrected values <0.017 (0.05/3) survive the correction and are considered significant.

#### Post-hoc lobule X specificity analyses

To determine whether this connectivity relationship was specific to subregions of the cerebellum or represented overall cerebellar function, we replicated the analysis with the control seed region unrelated to social functioning: lobule X (a region heavily implicated in vestibular function). As expected, this purely motor seed (lobule X) did not relate to any of the social deficits measured.

## Discussion

Individuals at CHR for psychosis showed broad social deficits across three domains: social interaction, cognition, and functioning. Though the cerebellum has been widely and consistently shown to be a site for social impairment in other clinical disorders [e.g., ASD; for a review see (28)], this study is the first to explore its contribution to social impairments in individuals at CHR for psychosis. Results from this study implicate the cerebellum as a critical neural correlate for social functioning among individuals at CHR for developing a psychotic disorder. Lower connectivity between the posterior cerebellum seed, lobule VIIIb, and the right frontal pole was related to poorer social functioning in the CHR for psychosis group. In contrast, higher connectivity between these regions in the control group related to superior social functioning. Importantly, to increase the specificity of these results, we found that a cerebellar subfield unrelated to social domains (e.g., lobule X) did not show these connectivity patterns. These findings indicate that individuals at CHR for developing a psychotic disorder may fail to engage cerebello-cortical connections necessary for supporting smooth and successful social experiences.

Social functioning impairments among individuals at CHR for developing a psychotic disorder were related to higher connectivity between lobule VIIIb and the right frontal pole compared to controls. Both these regions have been heavily implicated in decision making, monitoring and updating values, action observation, and attentional faculties (63, 68). Particularly within healthy controls, functional imaging studies have shown VIIIb to be strongly implicated in cognitions required for successful social functioning such as phonological memory and verb generation (69). And, evidence from morphological analyses done in autistic children found that decreased gray matter volume in this area significantly related to worse scores on social and communication items (27). This measurement of social functioning in this sample captures the most breadth of social processing since it holistically evaluates the quality and quantity of an individual's relationships. As such, the higher connectivity between these regions reflects a global impairment within the CHR group, whereby they may not be taking advantage of other efficient neural mechanisms to facilitate and maintain smooth relationships.

Connectivity results for the other measurements of social ability (social interaction quality and social cognition) were only at the trending level after additional corrections (see Supplemental material for results). These trending results merit a brief discussion given the that the potential social contributions of the cerebellum have been underexplored in this population. Resting-state social interaction impairments among individuals at CHR for developing a psychotic disorder were associated with lower connectivity between lobule VIIIa and the left precentral gyrus, whereas healthy controls exhibited higher connectivity. Lobule VIIIa is implicated in attentional resources and secondary motor representations (63, 70), while the left precentral gyrus is most commonly associated with voluntary movements. A potential explanation that warrants further study is that the motoric information likely relayed in the connectivity between lobule VIIIa and the precentral gyrus is distinctly social-motor information such as identifying and interpreting social movements and/or identifying facial expressions (71). At the trending level, impairments in social cognition among individuals at CHR for psychosis had higher connectivity between crus II and lobule VI. In comparison to control groups, individuals with schizophrenia have been shown to have higher intracerebellar connectivity (41), which may reflect impaired and uncoordinated internal models of social representations within those at clinical high risk for psychosis. Given that a primary function of the cerebellum is to improve motor, cognitive, and affective predictions, impairments can have a cascading effect on the quality and smoothness of how actors engage in the world.

Altered cerebrocerebellar connectivity has been widely observed in both schizophrenia populations and clinical high risks groups (36, 40, 72). Taken together, findings from the current study join the extant experimental work supporting evidence for Andreasen et al. (73–75). “cognitive dysmetria theory of schizophrenia” which posits that dysfunction in cerebello-thalamo-cortical circuitry results in mental incoordination, which give rise to heterogenous psychotic symptoms (36, 40, 72). The increased cerebrocerebellar connectivity patterns in the CHR group reflect potential mechanistic impairments that are present prior to the potential onset of a frank psychotic disorder.

Our results provide key new findings implicating the cerebellum as a neural correlate of social processing impairments among individuals at CHR for developing a psychotic disorder; however, some limitations need to be addressed. Although the sample size is comparable to other neuroimaging studies in this population [for a meta-analysis see (76)] there is slight variation in sample size between neuroimaging and social measures. Therefore, future efforts should aim to replicate these analyses in larger samples of this population (e.g., multisite consortium studies). In addition, the resting state scan time was limited to under 6 min to accommodate the reduced scanning tolerance of adolescents and those at CHR for psychosis. The length of the resting state scan is similar to work from other groups, particularly within this population, and has been shown to be equivalent power to longer scans (72, 77–79). The clinical high-risk state is highly heterogenous, and while some individuals may go on to convert to a psychotic episode, others experience stabilized CHR for psychosis symptoms, and some may experience fully remitted symptoms. Thus, distinct contributions of the cerebellum to social impairments may be important to consider within the context of clinical outcome. Future studies should look across the psychosis spectrum to improve our understanding of the nature and contribution of the cerebellum to social impairments. Additionally, cerebellar neuromodulation has been shown to be a promising treatment target within subclinical psychosis populations and future work should explore its potential to improve social deficits within the CHR for psychosis population. Gupta et al. (80) found improved motor learning rates within subclinical individuals following anodal cerebellar tDCS. Target parameters for cerebellar tDCS are variable with mixed findings, future work could utilize social cerebellar subregions as potential non-motor targets (81). While the current study did not have an extensive social processing battery, it included interrelated levels of social functioning across distinct informants. Despite the presence of these limitations, the findings in this current study identify a critical neural correlate to early social impairment symptoms in the high-risk for psychosis period, particularly within the context of null results in the control lobule. Importantly, social impairment is a transdiagnostic hallmark of many clinical and neurodevelopmental disorders beyond the psychosis spectrum [e.g., autism spectrum disorder (ASD), depression, bipolar disorder]. Disentangling the shared and distinct pathophysiology underlying these social impairments across these disorders is critical to elucidate the distinct etiologies and design effective interventions. Thus, future analyses of social impairments should include and pay particular attention to potential cerebellar contributions.

## Data availability statement

The original contributions presented in the study are included in the article/Supplementary material, further inquiries can be directed to the corresponding authors.

## Ethics statement

All procedures were approved by the Human Research and IRB University of Colorado, Boulder. Written informed consent to participate in this study was provided by the participants' legal guardians or next of kin.

## Author contributions

IRF, KSFD, and VAM conceptualized and wrote the initial draft. JAB provided feedback and contributed to the final draft. All authors contributed to the article and approved the submitted version.

## Funding

This work was supported by the National Institutes of Health (R01MH094650, R21/R33MH103231 to VAM) and National Institute of Mental Health (to KSFD). Details of all funding sources should be provided, including grant numbers if applicable.

## Conflict of interest

The authors declare that the research was conducted in the absence of any commercial or financial relationships that could be construed as a potential conflict of interest.

## Publisher's note

All claims expressed in this article are solely those of the authors and do not necessarily represent those of their affiliated organizations, or those of the publisher, the editors and the reviewers. Any product that may be evaluated in this article, or claim that may be made by its manufacturer, is not guaranteed or endorsed by the publisher.

## Footnotes

^1^To reflect the expressed preferences of many in the autistic community, we use identity-first language (“autistic individuals”) throughout the manuscript (22).
