# Supplementary material for: Cerebellar correlates of social dysfunction among individuals at clinical high risk for psychosis
Source: Front Psychiatry. 2022 Dec 1;13:1027470. doi: 10.3389/fpsyt.2022.1027470 (PMC9752902; doi:10.3389/fpsyt.2022.1027470)
Supplement: Supplementary file 1 [file Data_Sheet_1.docx]

Supplementary Material

# Relationship between social measurements

Correlation analyses were run to assess the nature of the relationship between the three social domains (Table 1). Among the HC group, there was no significant relationship among the three domains, whereas within the CHR group, there was a significant correlation between social functioning and social interaction quality, wherein high social functioning related to smoother social interaction qualities.

| Supplemental Table 1. Correlations between social processing domains by group | | | | |
| --- | --- | --- | --- | --- |
|  |  | Social Interaction | Social Cognition | Social Functioning |
| CHR | Social Interaction | 1 |  |  |
|  | Social Cognition | -.122 | 1 |  |
|  | Social Functioning | -.424** | .133 | 1 |
| HC | Social Interaction | 1 |  |  |
|  | Social Cognition | .244 | 1 |  |
|  | Social Functioning | -.292 | .46 | 1 |
| ** p<0.01  Note. Social interaction refers to social interaction difficulties measured by the Autism-Tics, ADHD and other Comorbidities inventory (A-TAC; (Larson et al., 2010)) in which higher scores refer to increased social interaction impairment. Social cognition is quantified by the Managing Emotions subtest of the Mayer-Salovey-Caruso Emotional Intelligence Test (MSCEIT-ME; (Mayer et al., 2012)) higher scores reflect efficient social cognition. Social functioning scores were tabulated using the Global Functioning Scale – Social (GFS-S; Cornblatt et al., 2007), where higher scores indicate successful maintenance, quality, and quantity of social relationships. | | | | |

# Trending connectivity patterns in social processing by group

A Bonferroni correction was applied to control for the three social regions. Trending results were greater than 0.017 (0.5/3) but less than 0.05.

## *Social Interaction.* To assess the relationship between social interaction quality on cerebellar connectivity across CHR and HC groups, a mean-centered social interaction covariate was compared across groups to predict any connectivity effect of lobule VIIIa. With a Bonferroni correction, there was a trending group by social interaction quality interaction which related to lobule VIIIa connectivity with the left precentral gyrus cluster (*p_FDR_*=.028, Table 2). Lobule VIIIa-left precentral gyrus connectivity was not related to social interactions among the CHR group (*r*=-.25, p = .104), but related to higher connectivity between these regions in the HC group (*r*=.52, p=.007). While it did not survive the Bonferroni correction, there was also a trending group by social interaction quality interaction related to lobule VIIIa connectivity with middle frontal gyrus cluster (*p_FDR_*=.035). Lower connectivity between lobule VIIIa and middle frontal gyrus was associated with impaired social interactions among the CHR group (*r*=-.36, p=.015), while the opposite pattern emerged for the HC group (*r*=.47, p =.015).

## *Social Cognition.* To assess the relationship between social cognition on cerebellar connectivity across CHR and HC groups, a mean-centered social covariate was compared across groups to predict any connectivity effect on social cerebellum regions (lobules VIIa, VIIb, VIIIa, and VIIIb), bilateral Crus II) in separate models. After applying a Bonferrni correction, there was a trending interaction between group, social cognition, and Crus II connectivity with lobule VI (*p_FDR_*=.04). Specifically, impaired social cognition was associated with lower resting-state connectivity between Crus II and lobule VI in the CHR group (*r*=.31, *p*=.015). The HC group revealed the opposite effect: impaired social cognition was associated with higher resting-state connectivity between Crus II and lobule VI (*r*=-.40, p<.001). The control seed region, lobule and X, and other posterior seeds were also investigated and did not result in any significant or trending findings.

| Supplemental Table 2. Cerebellar seed to voxel connectivity analyses - trending | | | | | | |
| --- | --- | --- | --- | --- | --- | --- |
|  | Coordinates | | |  |  |  |
|  | *x* | *y* | *z* | *cluster size* | *p_FDR-corrected_* | *p_uncorrected_* |
| Social interaction |  |  |  |  |  |  |
| VIIIa-left precentral gyrus | -44 | 10 | 34 | 123 | .02 | .002 |
| VIIIa-frontal gyrus | -52 | -02 | 48 | 96 | .03 | .02 |
| Social cognition |  |  |  |  |  |  |
| Crus II-lobule VI | -06 | -62 | -14 | 140 | .04 | .001 |
| *Note. A Bonferroni correction was applied to control for the three social regions. Only FDR-corrected values less than 0.017 (0.05/3) survive the correction and are considered significant.* | | | | | | |
